# Positive Humor and Work Withdrawal Behaviors: The Role of Stress Coping Styles in the Hotel Industry Amid COVID-19 Pandemic

**DOI:** 10.3390/ijerph19106233

**Published:** 2022-05-20

**Authors:** Ibrahim A. Elshaer, Alaa M. S. Azazz, Sameh Fayyad

**Affiliations:** 1 Department of Management, College of Business Administration, King Faisal University, Al-Ahsaa 380, Saudi Arabia; 2 Hotel Studies Department, Faculty of Tourism and Hotels, Suez Canal University, Ismailia 41522, Egypt; sameh.fayyad@tourism.suez.edu.eg; 3 Department of Tourism and Hospitality, Arts College, King Faisal University, Al-Ahsaa 380, Saudi Arabia; 4 Tourism Studies Department, Faculty of Tourism and Hotels, Suez Canal University, Ismailia 41522, Egypt

**Keywords:** hotels industry, affiliative, self-enhancing, coping styles, work withdrawal behavior, COVID-19

## Abstract

Working in the hospitality industry is stressful due to the intensive workload and extended work hours; this stress has increased after the COVID-19 pandemic due to high levels of job insecurity, downsizing, and laying off procedures. Employees in the hotel industry can deal with stress positively by utilizing task-coping styles or negatively by emotion- and avoidance-coping styles. Building on the transactional theory of stress and coping, and the benign violation theory of humor, the current study explores the relationships between positive humor and work withdrawal behaviors with the mediating effects of coping styles. A total of 407 hotel employees participated, and the obtained data were analyzed by structural equation modeling with partial least squares (PLS). The results asserted that affiliative humor is able to reduce coping with stresses via the negative styles and to increase coping with stresses via the positive style. The results also demonstrated the ability of task-coping in reducing work withdrawal behavior. Significant insights into theoretical and practical implications are further discussed.

## 1. Introduction

The ancient Egyptian deity Bes was associated with humor [1]. Egyptians are well known as awlād al-nukta (sons of the joke) for their adoration for jokes and ability to laugh even in hard times. Employees in the tourism industry are frequently stressed due to the intensive workload, extended working hours, and pressure to preserve a good image through high-quality work within a short period [2,3]. Amid the COVID-19 pandemic, the work pressure in hotels has increased due to layoffs, downsizings, and job and financial insecurity [4]. Employees’ work withdrawal behaviors—including decreased willingness to work, poor work performance, tardiness, early departure, and high turnover—are the most critical consequences of this stressful work environment.

Meanwhile, it is difficult to eliminate all the pressures in the hotel working environment and, to address this issue, researchers have asserted that coping methods must be improved, because effective coping with workplace-related stress is significant for individual well-being and job performance [5]. Jung et al. [6] claimed that employees might respond differently when faced with the same stressful environment depending on their characteristics. They might deal with stress positively by utilizing task-coping styles or negatively with emotion- and avoidance-coping styles.

According to scholars and practitioners, humor is a valuable workplace behavior and a helpful element for managers [7]. Frank A. Clark also said that “I think the next best thing to solving a problem is finding some humor in it”. Humor can help employees cope positively by changing their view of the stressors such that they see the pressure as benign and, in their mentality, recover a sense of control over any stress [8].

Building on the Egyptians’ sense of humor, transactional theory of stress and coping [9], and benign violation theory (BVT) [10] of humor, the current study aimed to explore the relationships among positive humor (affiliative humor and self-enhancing humor) stress coping styles, namely task-, emotion-, and avoidance-coping and work withdrawal behaviors, and to verify the mediating effects of coping styles on this relationship model. Despite its importance, research that has considered the associations between humor, stress-coping styles, and employees’ work withdrawal behaviors in hotels remains very rare.

## 2. Theoretical Background and Hypotheses Development

### 2.1. Humor in the Workplace

Researchers began to investigate the possibility that a sense of humor may contribute to workplace effectiveness in the 1980s [11]. Humor is defined as anything people think, say, or do that could be perceived as amusing and causes people to laugh [12]. Humor, as a social phenomenon, is complex and multifaceted and may manifest in a variety of ways, including jokes, anecdotes, funny stories, laughter, wit or satire, ironic phrases, or sarcasm [13].

Martin et al. and Dhensa-Kahlon et al. [14,15] categorized humor styles, based on their functions, into two adaptive types—affiliative and self-enhancing humor—and two maladaptive styles—aggressive and self-defeating humor. Affiliative humor uses jokes in a courteous, friendly manner to promote social cohesion. Self-enhancing humor entails keeping a good-natured and humorous mood in stressful or otherwise unfavorable situations. Aggressive humor relies upon insulting others; defeating humor indicates behaving comically or/and saying depreciated things about oneself to enhance interpersonal relationships.

According to social information processing theory [16], individuals interact with their workplace environment by observing social signals that surround them. Therefore, humor can send important social cues to employees to stimulate positive behaviors and hinder negative behavior in the work environment [17]. Positive humor is highly beneficial to workers in general. Studies have displayed that humor boosts performance, enhanced workplace communication, and strengthens relationships [8]. In their meta-analysis, Mesmer-Magnus et al. [11] discovered that positive workplace humor alleviates monotony and dissatisfaction and may have the potential to mitigate the negative impacts of workplace stress by acting as a coping mechanism (promoting relaxation, tension reduction, and dealing with disappointments), as well as its ability to lubricate social relationships in stressful situations. Yang et al. and Vecchio et al. [18,19] further argued that positive humor decreases employees’ work withdrawal behavior and turnover intentions. Moreover, the results of the study [20] found that aggressive humor can enhance a hotel’s knowledge-sharing culture if there is trust among employees.

### 2.2. Positive Humor and Stress Coping Styles

Coping styles refer to cognitive and behavioral strategies that people use to acclimate to perceived internal and/or external stressful situations [21,22]. Stress-coping styles are classified into positive and negative types [23]. The task-coping style represents the positive type. Employees use task-coping to take active and positive actions to change a stressful situation by creating positive appraisals and discovering solutions to resolve the problem [24]. On the other hand, emotion-coping and avoidance-coping represent negative coping styles. The emotion-coping style directs employees to vent their anger to others when facing a stressful situation without striving to recognize the problem itself [25]. However, some researchers argued that the emotional-coping style may be required to happen before the task-coping style can be effective. Identifying and comprehending emotions aids in coping by positively reinterpreting these emotions, which leads to an effective planning and implementation [5]. In the avoidance-coping style, employees lead their efforts toward minimizing, denying, or ignoring a stressful situation through other activities or just hoping that it will disappear [26].

According to incongruity theories, a humorous response implies broadening an individual’s viewpoint on a stressful circumstance, resulting in more appropriate problem-solving and coping styles [27,28]. Employees may use humor to navigate difficult work situations and ease stressful events [29]. As such, humor may help employees adopt positive coping styles (task-coping) by lubricating stressful work situations and using it to restore energy and the personal resources required to cope with the stress of unethical behavior and frustrating events.

Superiority theory claims that humor arises from feelings of superiority over other people or one’s former position. Thus, employees with a sense of humor feel mastery, self-esteem, and confidence that escort a humorous response to a stressful situation [30].

Warren et al. [10] recently proposed and tested a theory regarded as the strongest and most logical theory of how humor works. Warren et al. [10] benign violation theory (BVT) of humor proposed that humor can help employees consider the threat or violation as benign or harmless. Thus, it helps them to cope positively with stress.

**Figure 1:**
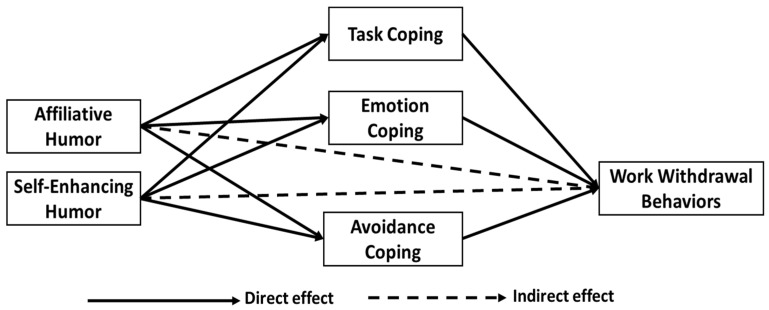
The proposed conceptual framework and hypotheses.

Based on the above debates, the current study hypothesizes that positive humor styles (affiliative and self-enhancing humor) affect task-coping styles (positive stress coping) positively and affect emotion-coping and avoidance-coping styles (negative stress coping) negatively. Thus, as pictured in Figure 1, these arguments direct to the following hypotheses:

**Hypothesis** **1** **(H1).** *Affiliative humor is positively related with the task-coping style.*

**Hypothesis** **2** **(H2).** *Affiliative humor is negatively related with emotion-coping.*

**Hypothesis** **3** **(H3).** *Affiliative humor is negatively related with the avoidance-coping style.*

**Hypothesis** **4** **(H4).** *Self-enhancing humor is positively related with the task-coping style.*

**Hypothesis** **5** **(H5).** *Self-enhancing humor is negatively related with emotion-coping.*

**Hypothesis** **6** **(H6).** *Self-enhancing humor is negatively related with the avoidance-coping style.*

### 2.3. Stress Coping Styles as a Mediator in the Relationship between Positive Humor Styles (Affiliative and Self-Enhancing Humor) and Employees’ Work Withdrawal Behaviors

Work withdrawal is defined as an employee’s avoidance and disengagement from their workplace [31]. Employees engage in work withdrawal behaviors when they become physically and/or psychologically disengaged from the organization where they work [32]. Work withdrawal behaviors, such as undesired work breaks, lateness to work, absenteeism from work, delay in doing work, lack of devotion to the job, and lack of organizational commitment [33], can, in addition to direct financial expenditures, reduce coworkers’ morale and motivation, damage team functioning, and result in eventual voluntary turnover [34].

Transactional theory of stress and coping demonstrates that employees may display adaptive coping or maladaptive coping behaviors when faced with workplace stressors [2]. According to the conservation of resources (COR) theory, subordinates may use work withdrawal behaviors as a negative coping tactic for stressful situations by doing bad work against the interests of their associations and causing counterproductive work behavior to maintain their depleted psychological and emotional resources [33,35]. Nevertheless, according to relief theory, humor enables individuals to adopt a positive coping style (task-coping) to offer an outlet to lessen negative behavior such as work withdrawal behaviors [36]. Based on the above arguments, it has been hypothesized that:

**Hypothesis** **7** **(H7).** *Task-coping style is negatively related with work withdrawal behaviors.*

**Hypothesis** **8** **(H8).** *Emotion-coping style is positively related with work withdrawal behaviors.*

**Hypothesis** **9** **(H9).** *Avoidance-coping style is positively related with work withdrawal behaviors.*

**Hypothesis** **10** **(H10).** *Coping styles mediate the relationship between affiliative humor and work withdrawal behaviors.*

**Hypothesis** **11** **(H11).** *Coping styles mediate the relationship between self-enhancing humor and work withdrawal behaviors.*

## 3. Methodology

### 3.1. Instrument Measurement

A self-administrated questionnaire was designed and developed to test the study hypotheses. An extensive review of the literature was employed to operationalize the study’s scales. This process yielded six dimensions. The affiliative humor (*a* = 0.932) and self-enhancing humor (*a* = 0.910) were tested by 16 items based on the Humor Styles Questionnaire (HSQ) scale [14]. The stress coping styles were measured using the nine-item scale proposed by Matthews et al. [37] as shown in Table 1. Finally, six items from Hanisch et al. [38] were employed to measure employees’ work withdrawal behaviors as a variable (*a* = 0.897). A Likert scale of 1 (strongly disagree) to 5 (Strongly agree) was employed. Six academics and six consultants tested the instrument for face and content validity. No changes were made to the questionnaire content, and it was read and clarified.

### 3.2. Participants and Data Collection

The research team distributed a total of 600 questionnaires. The study team comprises individuals who work in tourism and hotel management schools. As a result, they have a good relationship with human resources managers and general managers in the study’s selected hotels, who helped them collect data from customer-contact employees at hotels using a convenient sample and drop and collect methods in Sharm El-Sheikh (located in Egypt) during October 2021. Sharm El-Sheikh was chosen as it has many five-star hotels. Employees with at least three years’ experience were allowed to answer the survey as they have enough experience to answer the required questions. In total, 139 out of the 600 questionnaires were eliminated due to incomplete answers, leaving a total of 407 valid samples with a recovery rate of 68%. Respondents were required to sign a consent form, were given the option of accepting or declining the survey, and were informed that their responses would remain anonymous. This sample consisted of 76.7% males and 23.3% females between the age of 24 and 56 years (77%) as depicted in Table 1. The unmarried (30%) were fewer than the married (70%) employees. The majority of participants (79%) held bachelor’s degrees. Further, most respondents (95%) were Egyptian, while only 5% were non-Egyptian (usually working in the public relations department or animations department). More than half (55%) of the employees who participated in the study survey have working experience of more than 6 years, while 45% have working experience between 3 to 6 years.

An independent t-test sample technique was utilized to examine non-response bias and responding sample representativeness. The mean differences result of early and late responses showed no significant statistical value (*p* > 0.05), indicating that bias of non-response is not a concern in this study [39].

### 3.3. Data Analysis Methods

The present study utilized “Structural Equation Modeling” (SEM) with “Partial least squares” (PLS) technique to examine the hypotheses with Smart PLS-3 program. The suggested theoretical model was analyzed using a two-step approach (outer measurement model and structural model) as suggested by Leguina et al. [39].

## 4. Results of the Data Analysis

### 4.1. Assessment of Outer Measurement Model

To evaluate the outer model’s reliability and validity, the internal consistency reliability, indicator reliability, convergent validity, and discriminant validity were all evaluated. First, as displayed in Table 2, the structures’ internal consistency reliability was tested with Cronbach’s alpha (α) ranging from 0.848 to 0.932 and the composite reliability (CR) ranging from 0.908 to 0.944, which indicates satisfactory CR and α values.

Second, indicators’ reliability was acceptable, as all loading values of the structure indicators were higher than 0.60. Third, convergent validity was evaluated by the average variance extracted (AVE) values exceeding the satisfactory value of 0.50 [39]. Finally, three criteria were implemented to assess the discriminant validity of the constructs. They were cross-loading, the Fornell–Larcker criterion, and the heterotrait–monotrait ratio (HTMT) [39]. As indicated in Table 3, the outer-loading for each latent variable (underlined) was higher than the cross-loading with other measurements.

As depicted in Table 4, the bolded numbers of the AVEs in the diagonals outperform the correlation coefficient between variables. Jiang et al. [34] suggested that HTMT scores should be below 0.90 to support discriminant validity. As depicted in Table 4, all the HTMT readings were acceptable and below the cutoff point of 0.90 (see Table 4); thus, the outer measurement model’s findings were sufficient to proceed further with the structural model’s test.

### 4.2. Assessment of the Structural Model

The hypotheses were then tested by a “structural equation analysis” (SQM). In particular, the model’s predictive capacity and the explanatory power were analyzed [40]. With the VIF values of the manifest indicators changing within less than 5, the multicollinearity of the structural model has been verified as inexistent. Next, Henseler et al. [41] indicated that the lower limit for the R2 values is 0.10. Therefore, the R2 values for the variables of task-coping (R2 = 0.532), emotion-coping (R2 = 0.431), avoidance-coping (R2 = 0.394), and work withdrawal (R2 = 0.674) are acceptable (Table 5). Moreover, the Stone-Geisser Q2 test indicates task-coping, emotion-coping, avoidance-coping, and work withdrawal values greater than zero (Table 4), indicating adequate predictive validity of the model [42,43]. Accordingly, enough predictive validity for the structural model was also confirmed.

**Figure 2:**
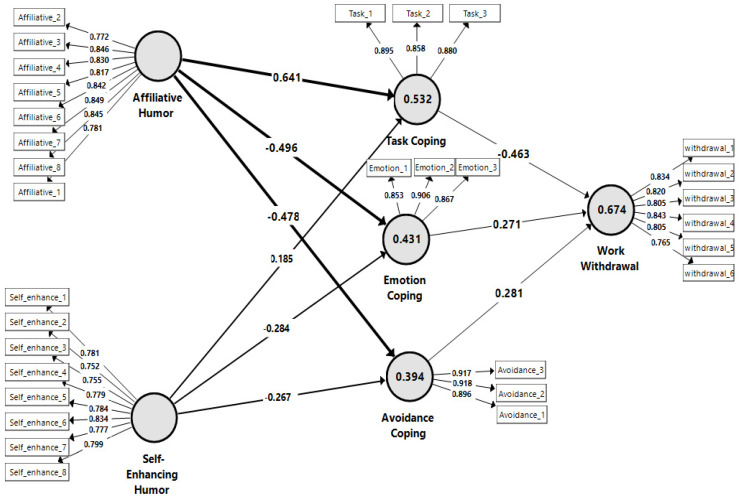
The tested structural and measurement model.

Lastly, the path coefficient and t-value of the hypothesized association were analyzed using a bootstrapping technique. Table 6 and Figure 2 below display the hypothesis test results, given the path coefficient values and the relevant significance. Affiliative humor was found to have a positive and significant correlation with task-coping at β = 0.641, *p* < 0.01, thus H1 was supported and has a negative and significant correlation with emotion-coping (β = −0.496, *p* < 0.01) and with avoidance-coping (β = −0.478, *p* < 0.01), supporting H2 and H3. The results showed that self-enhancing humor was positively related with task-coping at β = 0.185, *p* < 0.01, supporting H4, and it is negatively related with emotion-coping (β = −0.284, *p* < 0.01) and with avoidance-coping (β = −0.267, *p* < 0.01), supporting H5 and H6. The findings revealed that task-coping significantly and negatively influenced work withdrawal behaviors (β = −0.463, *p* < 0.01), supporting H7. Nevertheless, emotion-coping (β = 0.271, *p* < 0.01) and avoidance-coping (β = 0.281, *p* < 0.01) significantly and positively influenced work withdrawal behaviors, supporting H8 and H9. H10 posits that coping styles mediate the relationship between affiliative humor and work withdrawal behaviors. This was supported (β = −0.565, *p* < 0.01). Finally, self-enhancing humor has a negative effect on work withdrawal behavior through coping styles (indirect effect) at β = −0.237, *p* < 0.01, supporting H11.

## 5. Discussion and Implications

Overall, our findings indicate that positive humor helps employees to cope positively with stressful work situations, thus reducing employees’ work withdrawal behaviors. The empirical results revealed that affiliative humor and self-enhancing humor positively affect task-coping and have a negative effect on emotion-coping and avoidance-coping. This result is consistent with the incongruity theories that confirm that a humorous response implies broadening an individual’s viewpoint on a stressful circumstance, resulting in more appropriate problem solving and using positive coping styles (task-coping) [27]. These results further support the positive psychology’s perspective, where people with positive humor use it to positively cope with hard work situations and ease stressful events [29]. Martin et al. and Di Fabio et al. [14,44] confirmed that humor might be an automatic response akin to a defense tool rather than a consciously chosen approach to coping with stressful circumstances. In the same vein, Aldridge et al. [28] pointed out that the use of humor helps people become more adept at positively reframing stressful situations, leading to improved effects and psychological health, positive reframing, reversal, diminishment of stressful situations, and a reduction in maladaptive strategies. In other words, when individuals employ humor to aid them in coping, they usually find something about the stressor or the situation to laugh about. This helps them reappraise the stressor as less threatening, thereby relieving any stress encountered in the next appraisal [8].

Specifically, the results showed that affiliative humor, whether positive on task-coping or negative on emotion-coping and avoidance-coping, is more substantial than the impact of self-enhancing humor (as shown in Figure 2, bold lines for affiliative humor). This can be attributed to the fact that affiliative humor is used to enrich one’s relationships with others in a way that is relatively benign and self-accepting, whereas self-enhancing humor is centered internally and is used to help an individual to cope with stress [11].

Based on the results of empirical studies on the relationships among coping styles (task-, emotion-, and avoidance-coping) and work withdrawal behaviors, task-coping has a highly significant negative influence on work withdrawal behaviors, whereas emotion-coping and avoidance-coping have a significant positive effect on work withdrawal behaviors. This indicates that the more frequent the use of task-coping, the lower employees’ work withdrawal behaviors are, and vice versa with emotion-coping and avoidance-coping. This result agrees with the findings in Brittle et al. [45], which indicated that task-coping involves endeavoring to alter the events of stressful situations through problem-solving behaviors. On the contrary, the emotion-coping style directs employees to vent their anger to others when facing a stressful situation without striving to recognize the problem itself [25], and avoidance-coping refers to efforts to avoid or ignore a problem or stressor by withdrawing from stressful situations [46]. Therefore, Wilkinson et al. [47] assert that emotion-coping styles and avoidance-coping styles should not be considered coping strategies but rather risk factors for poor psychological health effects.

One of the study’s main aims was to examine the mediating role of stress coping styles (task, emotion, and avoidance) between positive humor (affiliative and self-enhancing humor) and employees’ work withdrawal behaviors. The study’s findings indicated that stress coping styles mediate the relationship between positive humor and employees’ work withdrawal behaviors. This pattern demonstrates how positive humor styles may have a valuable role in helping employees cope with stressors positively to reduce work withdrawal behaviors. This result can be explained by reference to the theory of benign violation theory (BVT), which suggests that there are conditions in which stressful situations may be viewed as benign rather than viscerally stressful. BVT ascribes this function to humor, changing how employees cope with otherwise stressful experiences, leading to reducing employees’ work withdrawal behaviors [8].

Based on the findings, the paper suggests that hotel practitioners and managers could take advantage of humorous features to improve their coping with stressful situations. According to social information processing theory [16], hotel managers can use a leader’s sense of humor to send critical social cues to subordinates to cope positively with a stressor, especially in times of crisis. In line with that, employees can reduce their work withdrawal behaviors with training to use positive humor and task-coping styles to face stressful work events.

## 6. Conclusions

The current study used the transactional theory of stress and coping and the benign violation theory (BVT) of humor to examine the relationships between positive humor (affiliative and self-enhancing humor), stress coping styles, specifically task-, emotion-, and avoidance-coping, and work withdrawal behaviors, as well as to verify the mediating effects of coping styles on this relationship model. Despite its significance, research on the relationships between humor, stress-coping styles, and employees’ work withdrawal behaviors in hotels is extremely rare. A total of 407 valid samples were collected and analyzed using SEM and Smart PLS program. Eleven hypotheses were proposed, and all were supported. The findings revealed that affiliative humor and self-enhancing humor both have a positive effect on task-coping while having a negative effect on emotion-coping and avoidance-coping. The researchers concluded that work withdrawal behaviors were found to be significantly better when task-coping was used, whereas work withdrawal behaviors were found to be significantly worse when using avoidance or emotion-coping. The findings of this study also revealed that stress coping styles is a mediating factor in the relationship between positive humor and employees’ work withdrawal behavior. This study is one of the few studies that focus on positive humor as a mechanism to enhance positive coping with stressors to reduce or eliminate employees’ work withdrawal behaviors in hotels. It is suggested that future research on humor may focus on humor as a leadership tool in hotels to improve their outcomes, especially during crises.

The study has some limitations that can be addressed by subsequent research. This study examined the effect of two types of humor (affiliative and self-enhancing humor) on work withdrawal behavior with the mediating role of coping styles (task-, emotion-, and avoidance-coping). However, there are several other dimensions such as job insecurity, distributive injustice, work intensification, and work environment, which may also impact work withdrawal behavior. They are not tested, however, in the current study. A broader range of mediating factors affecting the investigated relationships can be investigated in future research, and additional studies may employ some alternative research methodologies (e.g., qualitative research) to support and validate the current study’s findings.

The findings of the study were based on self-reported questionnaires, which may suffer from potential bias. As a result, future studies may collect data from different context (industry/country) and compare the results with those obtained from the current study. As the data are cross-sectional, it is difficult to establish a causal relationship between the variables studied. Applying the multi-group analysis method can also be used to compare the results in different contexts (i.e., industry or country).

## Figures and Tables

**Table 1:** The employees characteristics.

N = 1000		%	Groups	N = 407	%
**Age**			Egyptian	386	95
From 21 to 23	13	3	Non-Egyptian	21	5
24–40	122	30	Male	312	76.7
41–56	191	47	Female	95	23.3
More than 56	81	20			
**Marital status**					
Unmarried	122	30			
Married	285 200	70 20			
**Education level**					
High school level	321	79			
University level	86	21			
**Years of experience**					
3 to 5 years	183	45			
Over 6 years	224	55			

**Table 2:** Assessment of the formative measurement model.

Abbreviation	Outer Loading	α	C.R	AVE
**Affiliative humor**		0.932	0.944	0.678
Affiliative_1	0.78			
Affiliative_2	0.77			
Affiliative_3	0.85			
Affiliative_4	0.83			
Affiliative_5	0.82			
Affiliative_6	0.84			
Affiliative_7	0.85			
Affiliative_8	0.85			
**Self-enhancing humor**		0.910	0.927	0.613
Self_enhance_1	0.78			
Self_enhance_2	0.75			
Self_enhance_3	0.76			
Self_enhance_4	0.78			
Self_enhance_5	0.78			
Self_enhance_6	0.83			
Self_enhance_7	0.78			
Self_enhance_8	0.80			
**Task-coping**		0.851	0.910	0.771
Task_1	0.90			
Task_2	0.86			
Task_3	0.88			
**Emotion-coping**		0.848	0.908	0.767
Emotion_1	0.85			
Emotion_2	0.91			
Emotion_3	0.87			
**Avoidance-coping**		0.897	0.935	0.829
Avoidance_1	0.90			
Avoidance_2	0.92			
Avoidance_3	0.92			
**Work withdrawal**		0.897	0.921	0.660
withdrawal_1	0.83			
withdrawal_2	0.82			
withdrawal_3	0.81			
withdrawal_4	0.84			
withdrawal_5	0.81			
withdrawal_6	0.77			

**Table 3:** Cross loading results.

Abbreviation	Affiliative	Self-Enhancing	Task-Coping	Emotion-Coping	Avoidance-Coping	Work Withdrawal
Affiliative_1	**0.78**	0.30	0.49	−0.49	−0.46	−0.57
Affiliative_2	**0.77**	0.27	0.52	−0.43	−0.43	−0.53
Affiliative_3	**0.85**	0.31	0.60	−0.53	−0.50	−0.64
Affiliative_4	**0.83**	0.24	0.62	−0.49	−0.46	−0.65
Affiliative_5	**0.82**	0.26	0.57	−0.45	−0.44	−0.62
Affiliative_6	**0.84**	0.34	0.65	−0.50	−0.47	−0.73
Affiliative_7	**0.85**	0.41	0.61	−0.54	−0.49	−0.72
Affiliative_8	**0.85**	0.29	0.60	−0.53	−0.53	−0.69
Self_enhance_1	0.37	**0.78**	0.34	−0.36	−0.34	−0.40
Self_enhance_2	0.20	**0.75**	0.28	−0.34	−0.34	−0.37
Self_enhance_3	0.23	**0.76**	0.31	−0.36	−0.32	−0.34
Self_enhance_4	0.23	**0.78**	0.30	−0.33	−0.29	−0.36
Self_enhance_5	0.32	**0.78**	0.33	−0.33	−0.30	−0.40
Self_enhance_6	0.38	**0.83**	0.40	−0.46	−0.46	−0.46
Self_enhance_7	0.32	**0.78**	0.34	−0.37	−0.36	−0.37
Self_enhance_8	0.23	**0.80**	0.33	−0.35	−0.34	−0.34
Task_1	0.66	0.40	**0.90**	−0.45	−0.40	−0.65
Task_2	0.60	0.37	**0.86**	−0.40	−0.42	−0.57
Task_3	0.61	0.35	**0.88**	−0.35	−0.38	−0.66
Emotion_1	−0.46	−0.35	−0.35	**0.85**	0.35	0.46
Emotion_2	−0.60	−0.44	−0.46	**0.91**	0.45	0.61
Emotion_3	−0.51	−0.42	−0.38	**0.87**	0.47	0.54
Avoidance_1	−0.47	−0.43	−0.37	0.43	**0.90**	0.55
Avoidance_2	−0.53	−0.40	−0.44	0.43	**0.92**	0.55
Avoidance_3	−0.57	−0.39	−0.44	0.47	**0.92**	0.60
withdrawal_1	−0.68	−0.41	−0.60	0.53	0.54	**0.83**
withdrawal_2	−0.63	−0.43	−0.55	0.56	0.54	**0.82**
withdrawal_3	−0.65	−0.43	−0.58	0.49	0.50	**0.81**
withdrawal_4	−0.67	−0.35	−0.58	0.52	0.56	**0.84**
withdrawal_5	−0.62	−0.41	−0.61	0.47	0.46	**0.81**
withdrawal_6	−0.57	−0.35	−0.57	0.45	0.44	**0.77**

**Table 4:** Inter-construct correlations, the square root of AVE, and HTMT results.

	AVEs Values						HTMT Results					
	1	2	3	4	5	6	1	2	3	4	5	6
1. Affiliative humor	**0.823**						0.443					
2. Self-enhancing humor	0.490	**0.783**					0.370	0.525				
3. Task-coping	0.552	0.410	**0.878**				0.602	0.440	0.558			
4. Emotion-coping	−0.620	−0.527	0.557	**0.876**			0.530	0.324	0.507	0.556		
5. Avoidance-coping	−0.682	−0.631	0.137	0.337	**0.910**		0.662	0.431	0.337	0.537	0.370	
6. Work withdrawal	−0.499	−0.124	0.625	−0.424	−0.230	0.787	0.559	0.624	0.325	0.324	0.420	0.577

**Table 5:** Coefficient of determination (R2) and (Q2) of the model.

Endogenous Latent Construct	(R2)	(Q2)
Task-coping	0.532	0.31
Emotion-coping	0.431	0.31
Avoidance-coping	0.394	0.39
Work withdrawal	0.674	0.42

**Table 6:** The structural model’s results.

	Hypotheses	Beta (β)	(*T*-Value)	*p*-Values	Results of Hypotheses
H1	Affiliative humour → Task-coping	0.641	15.057	0.000	Accepted
H2	Affiliative humour → Emotion-coping	−0.496	11.473	0.000	Accepted
H3	Affiliative humour → Avoidance-coping	−0.478	11.608	0.000	Accepted
H4	Self-enhancing humour → Task-coping	0.185	4.905	0.000	Accepted
H5	Self-enhancing humour → Emotion-coping	−0.284	8.493	0.000	Accepted
H6	Self-enhancing humour → Avoidance-coping	−0.267	6.432	0.000	Accepted
H7	Task-coping → work withdrawal	−0.463	10.654	0.000	Accepted
H8	Emotion-coping → work withdrawal	0.271	5.258	0.000	Accepted
H9	Avoidance-coping → work withdrawal	0.281	5.920	0.000	Accepted
H10	Affiliative humour → coping styles → work withdrawal	−0.565	15.013	0.000	Accepted
H11	Self-enhancing humour → coping styles → work withdrawal	−0.237	9.808	0.000	Accepted

## Data Availability

Data are available upon request from researchers who meet the eligibility criteria. Kindly contact the first author privately through e-mail.
